# Symptoms of Chronic Appendicitis

**Published:** 1915-12

**Authors:** Wade H. Walker

**Affiliations:** Wichita Falls, Texas


### Symptoms of Chronic Appendicitis.

BY WADE H. WALKER, M. D., WICHITA FALLS, TEXAS.

In chronic appendicitis, when there has been no definite acute attack, the question is more difficult to decide. The patient may have merely had what he calls "bilious attacks" or "colic" or "intestinal indigestion." The picture presented by a chronically inflamed appendix may so closely simulate to gall stones or peptic ulcer that the diagnosis can only be cleared up by exploration of both regions. As these two lesions frequently co-exist, the surgeon should never rest content after one region alone is explored, if there is any question of the diagnosis. The upper quadrant should be explored first through a small incision, splitting the right rectus near its lateral margin.

If any lesion is found to exist in the gall bladder or ducts, duodenum, pancreas or stomach, the incision can be enlarged and the necessary procedure carried out. If the exploration is negative, the small wounds are closed. If the branch of the intercostal nerves have not been divided and the muscle and aponeurosis are carefully sutured, the abdominal wall will not be weakened. The appendix is then exposed and removed through the customary incision. If an attempt is made to accomplish all this through one incision placed midway between the two regions, it often will prove successful. But only too frequently will it prove awkward and difficult to approach both regions, adding to the trauma and time of the operation. The after-effect on the strength of the abdominal wall is more harmful than that of the two smaller incisions.

The simulation of a right upper quadrant lesion by a chronic appendicitis is often proved by the disappearance of these symptoms after removal of the appendix.

The "intestinal indigestion" cases, with chronic constipation, flatulence and occasional colic and general poor health, will usually show at one time or another a little appendicular tenderness. When such cases fail to improve after carefully planned and executed conservative measures, and other conditions can be excluded, an appendectomy is frequently indicated.

Another indication for removal of the appendix is when, during the course of some other abdominal procedure, this organ is found to be diseased, kinked or twisted, and its removal will not unduly add to the operative risk. In repairing a right inguinal hernia, it is often easy to bring the cecum and appendix out through the internal ring. If this can be done without undue traction and there is no evidence of a pathological condition, the appendix should be removed.

If, however, much traction is required to hold the cecum and mesentery in the wound, they should be replaced and a separate incision made at the usual site.

Doctors, didn't you know that this old world has waited over 1900 years for you to learn that appendicitis should be treated surgically first, last, and I might say always; and that you are the judge that should render the verdict always in favor of surgery, and never in favor of those who are so out of date as to want to treat a case medically. I have intimated above that you are the judge in this particular case, as well as the jury; you are the one to do the execution; in fact, immediately after the examining trial, you should let the patient pay the penalty of not having his liberty taken from him, but merely a small insignificant portion of his anatomy removed or divorced, in order to get relief and to have his rightful and personal liberty in the name of "good health," peace of mind, a long and possibly a happy life.

*"Chronic appendicitis"* should always be tried and arraigned before a jury of doctors and *surgeons* that are not moral cowards, but who are willing to stand for what they think and know to be right from a surgical standpoint; and who will not stand back on what the public thinks, or what their fellow physicians might say should have been done. Do as one of our great statesmen always did, using his motto, "Be sure you are right and then go ahead," and you will always be on the right side of the question. I had rather any surgeon would remove my appendix a year or two too soon, when I knew it was not absolutely necessary in order to save my life, than for the best surgeon in the world to remove my appendix one day, yes, one hour, too late, and to have my friends to walk slowly behind my corpse and say, "Poor fellow, he had all done for him that could be done; he had doctor so and so, a wonderful surgeon by reputation, but, alas! he was called too late."

Such cases bring surgery into disrepute, and immediately cause the town or city and surrounding country to be turned against surgery.

I again entreat you as men of a most noble profession to be on the alert, learn the symptoms of appendicitis, and learn them well. Learn all you can about the chronic and acute cases, and when you are first called to see a case your very first impression will usually be correct. On the other hand, if you dilly-dally along and listen to a consultant who has cured many a case with salts, castor oil and calomel, you will no doubt become persuaded or overinfluenced not to operate, you will lose confidence in your own self, and will have to call a surgeon from afar off to reap the benefits of your hard toils and long study and rightful case.

